# Determination of Aflatoxin B_1_ in Grains by Aptamer Affinity Column Enrichment and Purification Coupled with High Performance Liquid Chromatography Detection

**DOI:** 10.3390/foods13050640

**Published:** 2024-02-20

**Authors:** Cong Ji, Xinyang Sun, Yong Fang, Peng Li

**Affiliations:** College of Food Science and Engineering, Collaborative Innovation Center for Modern Grain Circulation and Safety, Nanjing University of Finance and Economics, Nanjing 210023, China; jicong0728@126.com (C.J.); xinyang.sun@nufe.edu.cn (X.S.); fangyong10@nufe.edu.cn (Y.F.)

**Keywords:** aflatoxin B_1_, HPLC, aptamer, grain, purification

## Abstract

Aflatoxin B_1_ (AFB_1_) is a highly teratogenic and carcinogenic secondary metabolite produced by *Aspergillus*. It is commonly detected in agricultural products such as cereals, peanuts, corn, and feed. Grains have a complex composition. These complex components severely interfere with the effective extraction and separation of AFB_1_, and also cause problems such as matrix interference and instrument damage, thus posing a great challenge in the accurate analysis of AFB_1_. In this study, an aptamer affinity column for AFB_1_ analysis (AFB_1_-AAC) was prepared for the enrichment and purification of AFB_1_ from grain samples. AFB_1_-AAC with an AFB_1_-specific aptamer as the recognition element exhibited high affinity and specificity for AFB_1_. Grain samples were enriched and purified by AFB_1_-AAC, and subsequently analyzed by high performance liquid chromatography with post-column photochemical derivatization-fluorescence detection (HPLC-PCD-FLD). The average recoveries of AFB_1_ ranged from 88.7% to 99.1%, with relative standard deviations (RSDs) of 1.4–5.6% (*n* = 3) at the spiked levels of 5.0–20.0 μg kg^−1^. The limit of detection (LOD) for AFB_1_ (0.02 μg kg^−1^) was much below the maximum residue limits (MRLs) for AFB_1_. This novel method can be applied to the determination of AFB_1_ residues in peanut, corn, and rice.

## 1. Introduction

Aflatoxins (AFs) are difuranocoumarin metabolites, which are mainly produced by food fungi *Aspergillus*, particularly *Aspergillus flavus*, *Aspergillus parasiticus* and *Aspergillus nomius* [1]. Among these AFs, AFB_1_ is recognized as the most toxic and prevalent, widely present in corn, rice, nuts, peanuts, and oil [2]. Annually, significant quantities of grains are contaminated with AFB_1_ due to extreme weather conditions or improper storage practices, particularly in hot and humid environments conducive to the growth of *Aspergillus* fungi [3,4]. As the International Agency for Research on Cancer (IARC) has classified AFB_1_ as a Group I human carcinogen, many countries and regions have set maximum residue limits (MRLs) for AFB_1_ in grains [5,6]. For example, the European Union (EU) has set the maximum level of AFB_1_ at 5.0 μg kg^−1^ in maize or rice, and 2.0 μg kg^−1^ in other cereals. In China, the mandated maximum level for AFB_1_ ranges from 5.0 to 20 μg kg^−1^ in different cereals [7]. The presence of AFB_1_ raises numerous health concerns in humans and livestock, as it exhibits mutagenic, teratogenic, carcinogenic, neurotoxic, and immunosuppressive properties [8]. Therefore, there is an urgent need for the development of robust analytical methods for detection of AFB_1_.

Thus far, analytical methods based on chromatography, spectroscopy, and immunoassay have been developed and used to determine AFB_1_. The chromatographic methods include high performance liquid chromatography (HPLC) [9], thin-layer chromatography (TLC) [10], and liquid chromatography-mass spectrometry (LC-MS) [11]. The spectroscopic methods include fluorescence spectrophotometry [12], infrared (IR) spectroscopy [13], and chromatin interacting protein-mass spectrometry (Chip-MS) [14]. In addition to these chromatographic and spectroscopic methods, immunoassays and biosensors have emerged as rapid and convenient tools for AFB_1_ detection, utilizing various recognition elements such as antibodies, enzymes, cells, and molecularly imprinted polymers [15]. However, while these rapid methods enable quick identification of AFB_1_, instrumental analytical techniques remain essential for accurate quantitative detection.

HPLC is one of the most powerful analytical instruments for mycotoxin analysis because of its advantageous features, including good usability, low cost, and high sensitivity. However, rigorous sample pre-treatment is required for AFB_1_ determination with HPLC. In particular, grain samples containing a large amount of complex matrices pose a great challenge for accurate detection, unless effective extraction and separation steps are performed. Therefore, for the determination of AFB_1_ in grain samples, pre-treatments such as extraction, purification, and concentration are required to achieve separation of interfering components and to effectively enrich traces of the analyte. The common pre-treatment techniques currently available include liquid-liquid extraction (LLE) [16], solid phase extraction (SPE) [17], supercritical fluid extraction (SFE) [18], immunoaffinity column (IAC) [19], and molecularly imprinted polymer (MIP) [20]. Among these, IAC based methods are widely utilized for the analysis of AFB_1_ in grain samples, as they enable the efficient and rapid separation and concentration of AFB_1_ from samples, while facilitating the removal of matrix interferences. For example, Shen et al. [21] determined aflatoxins in raw peanuts using IAC as sample clean-up method followed by normal-phase HPLC-fluorescence detector (HPLC-FLD) analysis. Aliakbarzadeh et al. [22] verified a standard method based on IAC cleanup and HPLC-FLD analysis for determination of aflatoxins in peanut kernels; the limit of detection (LOD) of the method was found to be 0.15 μg kg^−1^ for AFB_1_. IACs are usually prepared by binding AFB_1_-specific antibodies to an activated solid phase. As the sample extract passes through the column, AFB_1_ in the extract is bound by the antibody and adsorbed on the column. However, the use of antibodies as recognition elements has many disadvantages, including high production costs and susceptibility to denaturation during elution [23], underscoring the need for alternative technologies to IAC.

Aptamers are oligonucleotide compounds comprised of 20–100 nucleotides with a complex tertiary or quaternary structure [24]. Aptamers have been developed that can detect targets in the micromolar range, which is comparable to antibodies. High affinity aptamers are isolated by systematic evolution of ligands by exponential enrichment (SELEX), a process that relies on cycles of ligand selection and amplification of sequence libraries [25]. Xu et al. [26] determined the high-resolution structure of AFB_1_–AF26 DNA aptamer by solution NMR spectroscopy. Furthermore, based on the complex structure, they revealed the molecular mechanism of the aptamer’s discriminatory recognition of AFB_1_. To date, several aptamers have been applied as recognition elements for AFB_1_ binding. For example, Fan et al. [27] constructed an electrochemical aptamer sensor for the sensitive detection of AFB_1_, with a LOD as low as 4.8 fg mL^−1^. Li et al. [28] developed a colorimetric sensor using gold nanoparticles coupled with AFB_1_ and ochratoxin A (OTA) aptamers, the LOD of AFB_1_ was 0.07 ng mL^−1^. De Girolamo et al. [29] prepared an aptamer-based SPE column for OTA purification in wheat samples prior to HPLC-FLD analysis, with a LOD of 23 pg g^−1^. Zeng et al. [30] prepared Fe_3_O_4_@SiO_2_–NH_2_ material with a core–shell structure. Using this material, they developed an aptamer-based magnetic solid-phase extraction method for the pretreatment of AFB_1_ in bean sauce samples; the recoveries of AFB_1_ ranged from 80.19% to 113.92%. In comparison with antibodies, aptamers exhibit several outstanding features, including simple large-scale chemical production, easy chemical modification, low cost, and resistance to denaturation [31,32]. Thus, they are promising alternatives for various analytical applications, including AFB_1_ detection. However, the use of aptamers in enriching and purifying AFB_1_ from grains remains relatively underexplored.

In the present study, a novel AFB_1_-AAC was developed for the enrichment and purification of AFB_1_ in grains. After coupling with an amino-modified AFB_1_ aptamer, AAC immobilization was realized through a covalent reaction between isocyanate and amino groups. The operation procedures of AFB_1_-AAC were optimized, and its reproducibility and specificity were evaluated and compared with AFB_1_-IAC. The proposed method of AFB_1_-AAC enrichment and purification coupled with HPLC detection was validated by standard quality control materials (QCMs), and applied for the analysis of AFB_1_ in peanut, corn and rice samples.

## 2. Materials and Methods

### 2.1. Reagents and Chemicals

The sequence of a known AFB_1_-specific DNA aptamer was obtained from the literature [33]. The AFB_1_-specific aptamer sequence—5′-NH_2_C_12_-AAA AAA GTT GGG CAC GTG TTG TCT CTC TGT GTC TCG TGC CCT TCG CTA GGC CCA CA-3′—was then synthesized by Sangon BioEngineering (Shanghai, China). CNBr-activated sepharose (particle size, 45–165 μm) was purchased from Chromsep Biotechnology (Qingdao, China). AFB_1_ standard (10 µg mL^−1^) was purchased from RomerLabs (Tulln, Austria). AFs mixture standard (AFB_1_, AFB_2_, AFG_1_ and AFG_2_, 1000 ng mL^−1^), OTA (10 µg mL^−1^), zearalenone (ZEN, 100 µg mL^−1^), and deoxynivalenol (DON, 100 µg mL^−1^) were all purchased from Tan-Mo Technology (Changzhou, China). QCMs of AFB_1_-contaminated corn and wheat were provided by Meizheng Bio-Tech (Rizhao, China). Methanol and acetonitrile were HPLC grade. All other chemicals were at least analytical grade and were used without further purification. Ultrapure water from a Millipore Milli-Q system was used throughout. The grain samples were collected from a local supermarket (Nanjing, China) and stored at 4 °C.

### 2.2. Instrumentation

HPLC analyses were conducted using an Agilent 1260 series liquid chromatography system equipped with a fluorescence detector (Agilent, Santa Clara, CA, USA). A photochemical reactor (Huaan Magnech Bio Tech, Beijing, China) was also used for enhanced photochemical derivatization (PCD) detection. Empty columns for the fabrication of AAC columns were procured from Biocomma (Shenzhen, China). Commercial IACs for aflatoxins and mycotoxins were obtained from Romer Labs (Washington, DC, USA). The C18 column (150 mm × 4.6 mm i.d., particle size 5 µm) for HPLC separation was sourced from Phenomenex (Guangzhou, China). The benchtop rotary evaporator for removing solvent was provided by Heidolph (Shanghai, China).

### 2.3. Preparation of AFB_1_-AAC

The aptamer was first immobilized onto the surface of CNBr-activated sepharose. Briefly, the aptamer was dissolved in reaction buffer (200 mM Na_2_HPO_4_, 5 mM MgCl_2_, pH 8.0), heated at 85 °C for 5 min, and subsequently left at room temperature for 30 min to activate the aptamer. Concurrently, 0.5 mL of CNBr-activated sepharose was pre-washed and activated. Subsequently, the activated aptamers with different amounts (0, 0.02, 0.04, 0.06, 0.08, 0.1, 0.2, 0.4, and 0.5 nmol) were coupled to the CNBr-activated sepharose at room temperature with overnight shaking. Following the coupling step, the above sepharose was washed once with 1 mL of Na_2_HPO_4_ (200 mM, pH 8.0) and then blocked to deactivate any remaining active sites using 0.5 mL of Tris-HCl (0.1 M, pH 8.0). Any remaining uncoupled aptamer was removed by washing alternately with 1 mL of acetic acid buffer (0.1 M acetic acid, 0.5 M NaCl, pH 4.0) and 1 mL of Tris-HCl buffer (0.1 M Tris-HCl, 0.5 M NaCl, pH 8.0) three times. After centrifugation, the supernatant was discarded. The obtained AFB_1_ aptamer-sepharose was resuspended in binding buffer (20 mM Tris-HCl, 140 mM NaCl, 5 mM KCl, 1 mM MgCl_2_, pH 7.4), and loaded onto an empty AC column. Lastly, the prepared AFB_1_-AAC was washed with 10 mL of binding buffer, and stored at 4 °C in 0.5 mL of binding buffer.

### 2.4. Operation of AFB_1_-AAC

Grain sample extraction procedure: grain samples (5 g) were first pulverized and sonicated with 20 mL methanol for 20 min. Subsequently, the resulting mixture was centrifuged at 6800× *g* for 10 min. The obtained supernatant was removed using a rotary evaporator and the pellet was then re-solubilized with 25 mL of loading buffer. The obtained sample extract was filtered using a 0.22 µm filter membrane for subsequent enrichment and purification.

Enrichment and purification procedure: 20 mL of the above sample extract was loaded onto the AFB_1_-AAC. Next, the AFB_1_-AAC was washed with 1 mL of loading buffer to remove nonspecifically adsorbed interfering substances. Finally, bound AFB_1_ was eluted with 1 mL of elution buffer (methanol-acetic acid elution buffer, 98:2, *v*/*v*). The collected eluent was subsequently injected into the HPLC-PCD-FLD system for analysis. All the solutions were driven through the AFB_1_-AAC by gravity flow (flow rate, approx. 1 mL min^−1^).

AFB_1_-AAC regeneration procedure: the AFB_1_-AAC was equilibrated with 10 mL binding buffer and stored at 4 °C for further use.

### 2.5. HPLC-PCD-FLD Analysis

The conditions for HPLC-PCD-FLD analysis were optimized as follows. Chromatographic separation was performed on a C18 column (150 mm × 4.6 mm i.d., particle size 5 µm) maintained at 30 °C. The separation procedure was performed using an isocratic eluent of methanol-water (50:50, *v*/*v*) as the mobile phase. To achieve optimal resolution, a flow rate of 1.0 mL min^−1^ was employed. The sample injection volume was set at 20 µL. The excitation and emission wavelengths of the fluorescence detector were 360 nm and 440 nm, respectively.

## 3. Results and Discussion

### 3.1. AFB_1_-AAC Working Principle

**Figure 1:**
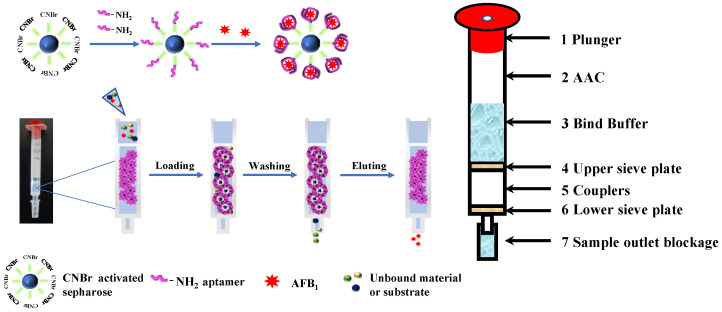
Schematic showing the production and use of AFB_1_-AAC. Top, conjugation of AFB_1_ and activated sepharose. Bottom, work flow for AFB_1_ purification on AFB_1_-AAC.

AFB_1_-specific aptamer was covalently linked to activated sepharose through a reaction between the -NH_2_ group of the amino modified aptamer and the -O-CN group of the CNBr-activated sepharose. The -NH_2_ group acts as a nucleophile, attacking the electrophilic carbon in the -O-CN group, leading to a stable amide bond formation. This reaction can easily occur at room temperature and is widely used in the field of biochemistry and molecular biology [34]. Detailed procedures for linking AFB_1_-specific aptamers with CNBr-activated sepharose, and the workflow for AFB_1_-AAC are illustrated in Figure 1.

The AFB_1_-AAC enrichment and purification procedure can be summarized in four steps: (1) column conditioning; (2) sample loading; (3) washing to remove interfering substances; and (4) elution of the target analyte. Prior to use, the AFB_1_-AAC is conditioned with 5 mL of loading buffer, equilibrating the column’s environment to optimize aptamer-AFB_1_ interaction. Following this, the AFB_1_-containing solution is introduced onto the column, where the aptamer’s unique three-dimensional structure and chemical properties facilitates selective binding of AFB_1_, distinguishing it from other molecules in the mixture. Subsequent washing removes non-specifically bound components, exploiting the differential binding affinities to ensure that only the target AFB_1_ remains adsorbed. Finally, the specifically bound AFB_1_ is released from the column using a carefully chosen elution reagent (methanol-acetic acid), which disrupts the aptamer-AFB_1_ interaction without damaging the integrity of either molecule. The eluted AFB_1_ is then collected for HPLC-PCD-FLD analysis.

### 3.2. Optimization of Operation Conditions of AFB_1_-AAC

**Figure 2:**
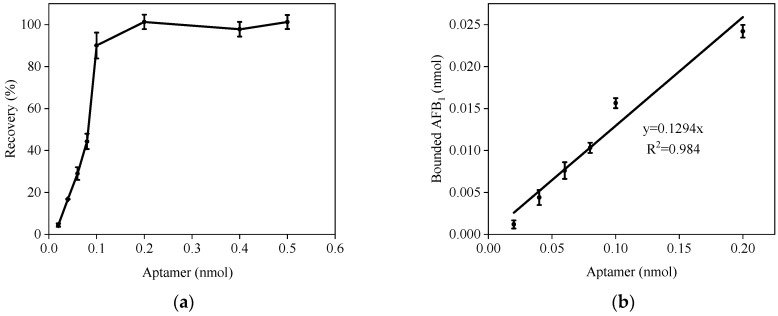
Effect of the immobilized aptamer on AFB_1_ recovery. (**a**) AFB_1_ recovery versus immobilized aptamer. (**b**) Aptamer molarity in relation to AFB_1_ (*n* = 3).

The amount of immobilized aptamer plays a decisive role in AFB_1_ capture. Various AFB_1_-AACs were prepared using differing quantities of aptamers to explore how aptamer dosage influences the recovery of AFB_1_. As depicted in Figure 2a, when these columns were treated with a 20 mL solution containing 5 ng of AFB_1_, it was found that AFB_1_ recovery improved with an increase in the aptamer amount from 0 to 0.1 nmol. When the amount of aptamer exceeded 0.2 nmol, the recovery rate increased to between 98.1% and 106.2%, meeting the requirements for quantitative recovery. These results also indicated that 0.2 nmol of immobilized aptamer can effectively bind approximately 0.016 nmol of AFB_1_.

To further elucidate the relationship between aptamer and AFB_1_, a standard curve was generated, with the molar amount of aptamer as the X-axis and the molar amount of bound AFB_1_ as the Y-axis. As shown in Figure 2b, the standard curve exhibited a slope of 0.13 and a correlation coefficient (R^2^) of 0.984. These results indicated that the amount of immobilized aptamer significantly influenced the enrichment and purification of AFB_1_. Thus, the amount of aptamer should be at least eight times that of AFB_1_.

**Figure 3:**
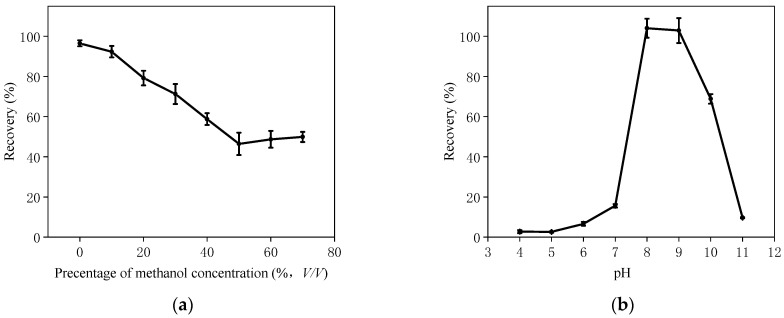
Effects of methanol concentration (**a**) and pH (**b**) of sample solution on AFB_1_ recovery (*n* = 3).

For grain sample analysis, the first step is extraction with solvent. In general, AFB_1_ is extracted from samples using organic solvents, such as pure methanol. However, organic solvents may impact the aptamer’s structure, potentially diminishing the affinity between the aptamer and its target [35]. Hence, organic components carried over from the extraction procedure may affect AFB_1_ capture on the AFB_1_-AAC. To address this issue, the maximum tolerance of the AFB_1_-AAC to methanol during the sample loading process was evaluated. First, the AFB_1_ standard solution was diluted with loading buffer containing varying concentrations of methanol (10%, 20%, 30%, 40%, 50%, 60%, and 70% (*v*/*v*)). Subsequently, an AFB_1_-AAC purification test was conducted. As shown in Figure 3a, AFB_1_ recovery decreased as the methanol concentration increased, indicating that the methanol concentration should be controlled below 10%. To ensure AFB_1_ retention on the column, the methanol in sample extract was evaporated using a rotavapor. Then, the sample was reconstituted with loading buffer to reduce the concentration of organic reagents and minimize the matrix interference.

The solution pH value has a significant impact on the affinity of an aptamer, as it directly affects the three-dimensional structure of the aptamer and its interactions with the target molecule [36]. If the pH value is too high or too low, it may lead to protonation or deprotonation reactions of the aptamer’s bases, altering their charge and consequently affecting the spatial structure of the aptamer. Such structural changes can weaken the binding affinity between the aptamer and the target molecule, or even completely inhibit their interaction [26]. Moreover, the structure and charge characteristics of the target molecule may also be affected by the pH value, further influencing the binding affinity of the aptamer [37]. Therefore, in the application of aptamers, researching and optimizing the impact of solution pH on aptamer affinity is crucial for achieving efficient and specific recognition. To address this issue, the pH tolerance of AFB_1_-AAC was evaluated by testing the AFB_1_ solutions with different pH (4.0–11.0). As depicted in Figure 3b, AFB_1_ recovery increased as the pH increased from pH 4.0 to pH 8.0, and then decreased drastically (from 104.1% to 9.7%) as the pH increased further from pH 9.0 to pH 11.0. Consequently, the optimal pH of the loading solution was set at pH 8.5 for this study.

In addition, the effect of flow rate on recovery was investigated at different flow rates in the range of 0.2−3 mL min^−1^. The average recovery for AFB_1_ exceeded 90% at each flow rate, showing that the variation in flow rate did not affect the recovery. Considering the convenience of use, the final design relied directly on natural gravity for operation, resulting in a solution flow rate of approximately 1 mL min^−1^.

### 3.3. Reusability of AFB_1_-AAC

**Figure 4:**
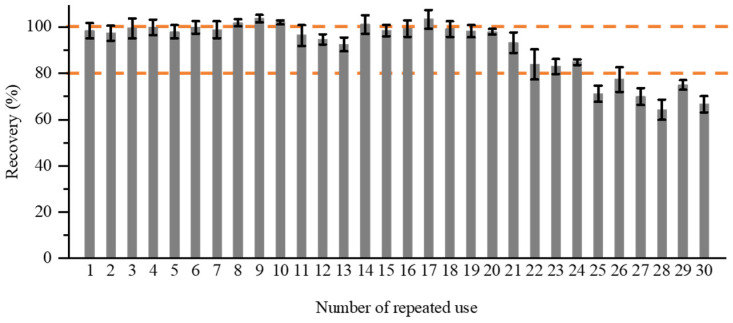
Effect of repeated use of AFB_1_-AAC on AFB_1_ recovery (*n* = 3).

The reusability of a column for sample pretreatment is a crucial factor in practical applications. The inability of IACs to be recycled is a major factor contributing to their high cost of utilization. The tolerance of aptamers to harsh conditions, including high salt and organic solvents, is known to be much higher than that of antibodies [38]. To evaluate the reusability of the prepared AFB_1_-AAC, it was tested over 30 cycles of loading, washing, and elution. As shown in Figure 4, during the initial 24 cycles, the average recovery of AFB_1_ ranged from 82.9% to 103.3%, and RSDs were within the range of 0.9–4.3%. However, the AFB_1_ recovery declined to 80% after the 24th cycle. These results demonstrate the favorable reproducibility of AFB_1_-AAC and its potential for cost-effective, repetitive usage.

### 3.4. Specificity of AFB_1_-AAC

**Figure 5:**
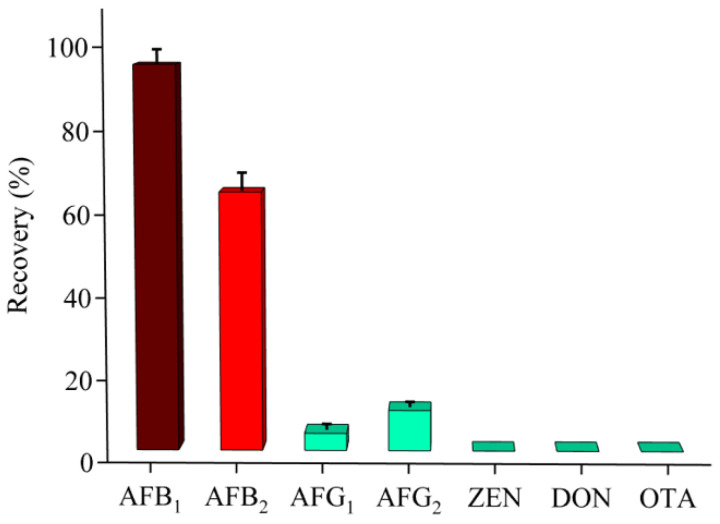
Recovery of various mycotoxins using the AFB_1_-AAC extraction procedure (*n* = 3).

In general, coexisting interfering substances in the solution may compete with the target analyte for active binding sites. To assess the specificity of AFB_1_-AAC, several AFB_1_ structural analogs—AFB_2_, AFG_1_, and AFG_2_—and several other toxins commonly found in grains—ZEN, DON, and OTA—were tested using the same procedure and conditions. As shown in Figure 5, the recoveries of AFG_1,_ AFG_2,_ ZEN, DON, and OTA were all very poor after AFB_1_-AAC extraction. However, the recovery of AFB_2_ was around 64.1%, suggesting that AFB_1_-AAC also possessed an affinity for AFB_2_. Nonetheless, due to the marked disparity in retention times between AFB_2_ and AFB_1_ on the HPLC-PCD-FLD chromatogram (7.13 min and 8.42 min, respectively), the specificity of AFB_1_ remained largely unaffected. Therefore, the presence of other mycotoxins in the grain does not affect the detection of AFB_1_ by AFB_1_-AAC. Besides, it is conceivable that with the selection of AFB_1_ aptamers with higher specificity, the possible for cross-reactivity could be further reduced.

### 3.5. Comparison of AFB_1_ Purification Using AFB_1_-IAC and AFB_1_-AAC

**Figure 6:**
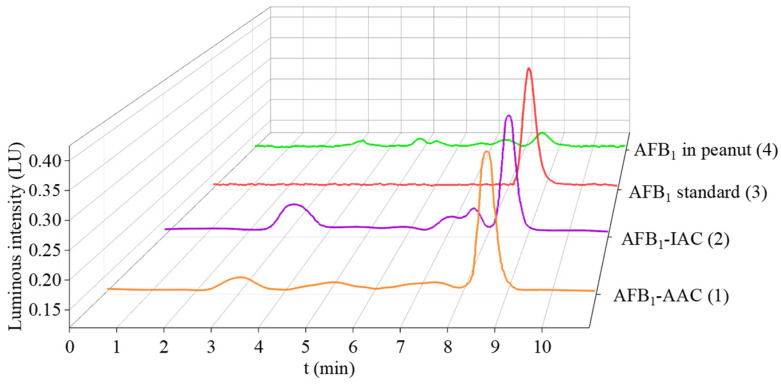
Comparison of AFB_1_ purification using AFB_1_-IAC and AFB_1_-AAC. Chromatograms of AFB_1_ contaminated peanut treated by AFB_1_-AAC (1) and AFB_1_-IAC (2), AFB_1_ standard solution in methanol (3), peanut extract without purification (4).

To evaluate the practical applicability of AFB_1_-AAC for the enrichment and purification of AFB_1_, it was compared with a commercial AFB_1_-IAC for the determination of AFB_1_ in peanuts. The resulting chromatograms are displayed in Figure 6. The results showed a significant intensification of the AFB_1_ peak, indicating that the target AFB_1_ was efficiently enriched using both the AFB_1_-AAC and the AFB_1_-IAC. In addition, both baselines were relatively clean. However, a meticulous comparison of the chromatograms of AFB_1_-IAC and AFB_1_-AAC revealed that there was less interference around the AFB_1_ peak by using AFB_1_-AAC. This suggested that AFB_1_-AAC may exhibit the greater tolerance to the complicated sample matrices, effectively removing interfering substances from sample extracts. Most importantly, there was no significant difference in the detected AFB_1_ between AFB_1_-AAC and AFB_1_-IAC extractions. Therefore, the AFB_1_-AAC can be confidently used as an alternative sample treatment method to AFB_1_-IAC.

### 3.6. Storage Stablility and Preparation Reproducibility of AFB_1_-AAC

The storage stability of the AFB_1_-AAC was assessed by evaluating its performance at three and six weeks post-preparation, with storage conditions maintained at 4 °C in a fridge. The recoveries of AFB_1_ consistently exceeded 90.0%, demonstrating the robust stability of the AFB_1_-AAC over time. Moreover, the preparation reproducibility of the AFB_1_-AACs was examined by testing the columns prepared within the same batch (*n* = 6) and across different batches (*n* = 6). The resulting RSDs for analyses of the same AFB_1_-contaminated sample were 3.4% for intra-batch and 4.6% for inter-batch comparisons, respectively. These low RSD values indicated that the AFB_1_-AACs were reliably reproducible, ensuring consistent performance in the detection of AFB_1_ in contaminated samples [39].

### 3.7. Method Validation and Application

Under optimized conditions of AFB_1_-IAC enrichment and purification, the LOD (calculated as a signal-to-noise ratio (S/N) of 3), and the limit of quantitation (calculated as a S/N of 10), were 0.02 µg kg^−1^ and 0.06 µg kg^−1^, respectively. Table 1 presents a comparison of the analytical performance obtained using the AFB_1_-AAC method with the previous works. The developed method for AFB_1_-AAC enrichment and purification in this study displayed good sensitivity and a high enrichment factor.

The developed method was validated by determination of AFB_1_ in QCMs. The analytical results are reported in Table 2. The good agreement between certified and determined values demonstrates the excellent accuracy of the proposed method. Based on the abovementioned findings, this successfully validated method was applied to the determination of AFB_1_ in peanut, corn and rice samples. The analytical results along with recoveries for the spiked samples are given in Table 3. The average recoveries of AFB_1_ in peanut, corn, and rice samples were 93.3–95.3%, 88.8–99.1%, and 88.7–95.0%, with RSDs of 1.4–3.0%, 1.8–5.6%, and 2.1–4.6%, respectively. These results demonstrate that the proposed AFB_1_-AAC enrichment and purification coupled with HPLC-PCD-FLD determination can be effectively used for the quantitative analysis of AFB_1_ in grain.

It is worth noting the construction of the AFB_1_-AAC offers significant benefits, including low raw material costs and an eco-friendly synthesis process. All the materials for the AFB_1_-AAC are readily available. According to the calculations based on quotations from the suppliers of raw materials, the manufacturing cost per AFB_1_-AAC would typically be less than USD $0.9, which is at least ten times lower than the IAC. Moreover, given its potential for reuse, the AFB_1_-AAC is economically acceptable and suitable for routine sample testing, which enhances its practicality in various applications.

## 4. Conclusions

In the present study, an AFB_1_-AAC for the enrichment and purification of AFB_1_ was prepared by covalently coupling an amino-modified AFB_1_-specific aptamer to CNBr-activated sepharose. The conditions for the use of this AFB_1_-AAC were optimized, and its reproducibility and specificity were investigated. The AFB_1_-AAC exhibited superior reproducibility, maintaining consistent performance over 24 repeated cycles. For spiked peanut, corn, and rice samples, the AFB_1_-AAC was able to remove matrix interferences and enable the quantitative analysis of AFB_1_. Comparison of AFB_1_-AAC with commercial AFB_1_-IAC also showed excellent results. AFB_1_-AAC exhibits the advantages of low cost, environmentally friendly attributes, and high reproducibility, and the proposed method can be used for the enrichment and separation of AFB_1_ in peanut, corn, and rice. Future efforts will focus on identifying AFB_1_ aptamers with superior performance and extending the method’s applicability to a broader range of food matrices.

## Figures and Tables

**Table 1:** Comparison of analytical performance of the proposed AFB_1_-AAC method with related previous methods.

Pretreatment Technique	Enrichment Factor	Determination Technique	LOD (µg kg^−1^)	Reference
SiO_2_@Fe_3_O_4_	5	HPLC-MS/MS	0.04	[40]
QuEChERS	5	LC-MS/MS	0.03	[41]
Immunoaffinity column	12.5	HPLC-PCD-FLD	0.03	[42]
HLB SPE cartridge	14	LC-ESI-MS/MS	0.017	[43]
AFB_1_-AAC	20	HPLC-PCD-FLD	0.02	This work

**Table 2:** Analytical results for determination of AFB_1_ in QCMs (mean ± SD, *n* = 3).

QCMs	Matrix	Certified (µg kg^−1^)	Found (µg kg^−1^)
MRM0021-0 (A101903B)	wheat	0.0 ± 0.0	ND
MRM0001-0 (A012001B)	corn	23.3 ± 3.5	21.3 ± 1.5
MRM0021-0 (A032303B)	wheat	33.2 ± 5.0	31.6 ± 1.4

**Table 3:** Recoveries of AFB_1_ in grain samples with different spiking levels.

	No.	Spiked (µg kg^−1^)	Recovery (%)	RSD (%, *n* = 3)
Peanut	1	5	93.3	2.6
	2	10	95.3	3.0
	3	20	94.2	1.4
Corn	1	5	99.1	1.8
	2	10	88.8	2.4
	3	20	91.4	5.6
Rice	1	5	94.3	3.7
	2	10	95.0	2.1
	3	20	88.7	4.6

## Data Availability

The original contributions presented in the study are included in the article, further inquiries can be directed to the corresponding author.
